# Supplementary material for: Majority of new patient referrals to a large pediatric rheumatology center result in non-rheumatic diagnosis
Source: Pediatr Rheumatol Online J. 2023 Oct 13;21:120. doi: 10.1186/s12969-023-00910-y (PMC10571278; doi:10.1186/s12969-023-00910-y)
Supplement: Supplementary file 1 — Supplementary Material 1 [file 12969_2023_910_MOESM1_ESM.docx]

Daniel D Reiff, MD

University of Alabama at Birmingham

Department of Pediatrics, Division of Rheumatology

1600 7th Ave S, CPPN G10

Birmingham, AL 35233

(205) 638-9438

danr2096@gmail.com

August 11, 2023

We are pleased to submit a full-length article titled “Majority of New Patient Referrals to a Large Pediatric Rheumatology Center Result in Non-Rheumatic Diagnosis” for consideration for publication in *Pediatric Rheumatology*

- This manuscript has not been published and will not be under consideration for publication elsewhere while submitted to *Pediatric Rheumatology*.

- If accepted to *Pediatric Rheumatology*, we will not seek publication elsewhere.

- The authors have no conflicts of interest to disclose relevant to this publication and no funding was received for this study.

- This manuscript was produced under a quality improvement initiative and as such does not require IRB approval, as it does not qualify as research.

- All authors have participated in concept/design, analysis/interpretation of data, and/or drafting or revising of this manuscript.

- All authors are responsible for reported research and approve of the manuscript as submitted.

- If accepted, we agree to bear the applicable publication charges as determined by *Pediatric Rheumatology*.

Thank you for your consideration.

Sincerely,


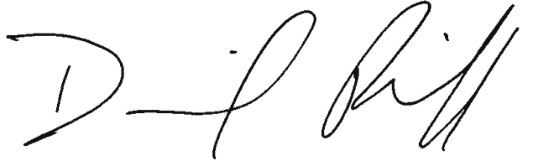


Daniel D Reiff, MD

Fellow, Pediatric Rheumatology

University of Alabama at Birmingham
