# Supplementary material for: Majority of new patient referrals to a large pediatric rheumatology center result in non-rheumatic diagnosis
Source: Pediatr Rheumatol Online J. 2023 Oct 13;21:120. doi: 10.1186/s12969-023-00910-y (PMC10571278; doi:10.1186/s12969-023-00910-y)
Supplement: Supplementary file 2 — Supplementary Material 2 [file 12969_2023_910_MOESM2_ESM.docx]

**Full Title:** Majority of New Patient Referrals to a Large Pediatric Rheumatology Center Result in Non-Rheumatic Diagnosis

**Authors:** Daniel D Reiff, MD^1, 2^; John M Bridges, MD, MS^1^; Eileen C Rife, MD, MPH^1^; Victoria L Gennaro, MSN, CPNP-PC^1^; Linda McAllister, MSN, PNP-AC^1^; Annelle Reed, MSN, CPNP-PC^1^; Carolyn Smith, MSN, CPNP-PC^1^; Bethany Walker, MSN, CPNP-PC^1^; Peter Weiser, MD^1^; Emily A Smitherman, MD, MS^1^; Matthew L Stoll MD, PhD, MSCS^1^; Melissa L Mannion, MD, MSPH^1^; Randy Q Cron, MD, PhD^1^

**Affiliations**:

^1^Division of Rheumatology, Department of Pediatrics, University of Alabama at Birmingham, Birmingham, AL, USA

^2^Department of Pediatric Rheumatology, Boys Town National Research Hospital, Omaha, NE, USA

**Correspondence and reprint requests to:**

Dr. Daniel D Reiff

Boys Town National Research Hospital

14040 Boys Town Hospital Rd

Boys Town, NE 68010

Phone: (531) 355-6800 Fax: (531) 355-0027 Email: daniel.reiff@boystown.org

**Abstract**

*Objective*

Pediatric rheumatology faces a looming supply-demand crisis. While strategies have been proposed to address the supply shortfall, investigation into the increased demand for pediatric rheumatic care has been limited. Herein, we analyze new patient visits to a large tertiary care pediatric rheumatology center to identify emerging trends in referrals and areas for potential intervention to meet this increased demand.

*Methods*

All patients referred to and seen by the University of Alabama at Birmingham Pediatric Rheumatology Division between January 2019 and December 2021 for a new patient evaluation were identified. Patient data was retrospectively abstracted, de-identified, and analyzed to develop trends in referrals and frequency of rheumatic disease, non-rheumatic disease, and specific diagnoses.

*Results*

During the study period, 2638 patients were referred to and seen in by the pediatric rheumatology division. Six hundred and ten patients (23.1%) were diagnosed with rheumatic disease. The most common rheumatic disease was juvenile idiopathic arthritis (JIA) at 45.6%, followed by primary Raynaud phenomenon (7.4%), recurrent fever syndromes (6.9%), vasculitides (6.7%), and inflammatory eye disease (6.2%). Of the 2028 patients (76.9%) diagnosed with a non-rheumatic condition, benign musculoskeletal pain was the most common (61.8%), followed by a combination of somatic conditions (11.6%), and non-inflammatory rash (7.7%).

*Conclusion*

In this analysis of new patient referrals to a large pediatric rheumatology center, the majority of patients were diagnosed with a non-rheumatic condition. As a worsening supply-demand gap threatens the field of pediatric rheumatology, increased emphasis should be placed on reducing non-rheumatic disease referrals.

**Introduction**

Since its emergence as a distinct pediatric subspecialty in the 1970s, pediatric rheumatology has become crucial in the management of children with complex and life-threatening diseases associated with organ and connective tissue inflammation (1). More recently, we have seen novel immunomodulatory therapies, targeted genetic testing, and expansion of international patient registries improve diagnosis, treatment, and outcomes for children with rheumatic disease. However, despite these advancements, a simultaneous contraction of the United States pediatric rheumatology workforce and increased demand for rheumatology evaluation threaten to overwhelm the system. The 2015 American College of Rheumatology Workforce Study projected a significant increase in the supply-demand gap for pediatric rheumatology care over the next 10-20 years due to many factors, including an aging pediatric rheumatology workforce, few fellow graduates, expansion of the overall pediatric population, and concentration of providers in academic centers (2). While strategies have been proposed to address the supply shortfall, there is limited data looking into the demand for rheumatic care at the level of individual centers (Correll ACR). The three most recent analyses of individual center and small collections of pediatric rheumatology clinic populations were reported in 1994, 1996, and 2005. In 1994, Denardo et al. prospectively enrolled 4585 new pediatric rheumatology patients from eight clinics in southern New England over an 8-year period, reporting their diagnoses and incidence of rheumatic disease (3). Then in 1996, Bowyer and Roettcher published on the diagnoses of a larger cohort of 12939 pediatric rheumatology patients from 25 clinics over a 3-year period (1992-1995) from across the United States (4). Lastly, in 2005, Rosenberg reported on diagnoses and disease frequencies of 3269 patients referred to the Pediatric Rheumatology Clinic at the University of Saskatchewan over a 23-year period (1981-2004) (5). Twenty years later, we aim to add to this knowledge by analyzing three years of new patient visits to a large tertiary care pediatric rheumatology center in order to identify emerging trends in referrals and areas for potential intervention to meet increased demand.

**Methods**

*Subjects and Referral Process*.

The study population includes all patients referred to and seen by the University of Alabama at Birmingham Pediatric Rheumatology Division between January 2019 and December 2021 for a new patient evaluation. All care was provided at Children’s of Alabama and associated satellite locations within the state. In alignment with department policy, all patients under the age of 18 referred for rheumatology evaluation were offered an appointment, regardless of suspicion for rheumatic disease during the referral triage process. Referrals come from providers within the Children’s of Alabama system, community advanced practice providers and pediatricians, and from surrounding states in the American Southeast. All referrals were reviewed by pediatric rheumatologists within the division upon receipt for determination of acuity. Referrals that did not result in an attended appointment, including cancellations and “no-shows”, were excluded from analysis, as an accurate determination of diagnosis was unable to be reached. Patients initially evaluated as inpatient consults, but subsequently followed in rheumatology clinic, were also excluded.

*Methods and Determination of Diagnosis.*

De-identified patient data was retrospectively abstracted from the electronic medical record system for the observable time between January 2019 and December 2021. Variables collected for each new patient included initial referral reason as per the referring provider, referral date, first appointment date, attended follow-up appointments, and final diagnosis. Diagnoses were assigned to a disease category via generally accepted rheumatic classification criteria or diagnostic assessments. If patients had their diagnosis changed at any point during their care, the final diagnosis or most recent diagnosis at the time of data abstraction was used in this analysis. A patient’s diagnosis was classified as a “rheumatic disease” if it requires chronic management primarily by or in conjunction with a pediatric rheumatologist. During the study period, one of six different pediatric rheumatologists primarily managed each patient, with assistance from nurse practitioners and fellows-in-training.

Data abstraction and analysis was undertaken as a Quality Improvement initiative within the University of Alabama at Birmingham Pediatric Rheumatology Division, with the goal to improve the appointment referral process and decrease appointment wait times. Given the specificity of the data to our individual center, the patient data used does not contribute to generalizable knowledge and this project therefore does not meet the formal definition of research per the US Department of Health and Human Services and was not formally supervised by the Institutional Review Board per policy. Analysis and calculations were performed with Microsoft Excel. Data was presumed to be non-normal in its distribution, so continuious variables were expressed in terms of median and interquartile ranges (IQR).

**Results**

Between January 2019 and December 2021, 2638 patients were referred to and seen by our pediatric rheumatology clinic. Of these patients, 610 (23.1%) were eventually diagnosed with a rheumatic condition (Table 1). After their initial evaluation, only 33% of new patients were seen for a follow-up appointment, including 82.8% of patients with rheumatic diagnoses and 18.0% of non-rheumatic conditions (Table 1).

On a month-to-month basis, excluding February 2020 through May 2020 when clinic was significantly limited during the onset of the coronavirus disease 2019 (COVID-19) pandemic, appointments ranged from 52-137 new patients seen monthly with a median of 79 new patients per month (IQR 68-86) (Figure 1). The number of new rheumatic disease diagnoses ranged from 11-26 monthly (median 18, IQR 14-21) and non-rheumatic diagnoses ranged from 36-116 (median 58, IQR 50-68). The median proportion of patients seen with a rheumatic diagnosis was 22.2% of patients per month, consistent with the overall proportion of 23.1% throughout the study period.

Of the 610 patients diagnosed with a rheumatic condition during the study period, the most common diagnosis was juvenile idiopathic arthritis (JIA) at 45.6% of diagnoses (Table 2). Oligoarticular JIA was the most prevalent subtype comprising 33.5% of JIA diagnoses, followed by enthesitis-related JIA (19.8%), psoriatic JIA (17.3%), and rheumatoid factor negative polyarticular JIA (15.1%). No other diagnosis group comprised greater than 10% of the population. The next most common diagnoses included primary Raynaud phenomenon (7.4%), recurrent fever syndromes (6.9%), vasculitides such as ANCA-associated vasculitis, Henoch-Schönlein purpura, and Kawasaki disease follow-up (6.7%), and inflammatory eye disease including uveitis (6.2%). Other diagnosis groups made up less than 5% of the total rheumatic disease population. The median time from referral to appointment for patients with a rheumatic disease diagnosis was 13.8 days (IQR 4.9-46.0), with all individual diagnosis wait times (except Raynauds phenomenon) under 28 days (Table 2).

Two thousand and twenty-eight patients were diagnosed with a non-rheumatic cause of their chief complaint during initial or follow-up evaluation (Table 3). Musculoskeletal pain was the most common non-rheumatic diagnosis, with 1253 (61.8%) patients diagnosed during the study period. Within the musculoskeletal pain category, 880 patients (43.4% of all non-rheumatic diagnoses) were diagnosed with musculoskeletal pain of a specific joint, followed by back pain and “other” musculoskeletal pain (e.g., “hand pain”, “foot pain”, etc.). Amplified musculoskeletal pain syndrome, chronic fatigue syndrome, and complex regional pain syndrome together made up 235 patients (11.6%), followed by non-inflammatory rash (7.7%) and recurrent fevers (5.9%). The “other” category totaled 117 patients (5.8%) with various diagnoses listed in Table 4. The median appointment wait time for patients with non-rheumatic diagnoses was found to be 49 days (IQR 20-69.9) with individual non-rheumatic diagnosis wait time ranging from 14.7 days to 84.0 days (Table 3).

**Discussion**

While national and international registries of pediatric rheumatology patients have grown over the last 10-20 years, analysis of individual center populations has been lacking in the literature. Although viewing the field of pediatric rheumatology through the lens of a single-center experience has limitations with respect to the advancement of treatment and diagnosis of rare diseases, it can shed a unique light on the supply-demand challenges facing the field today. Analyses by Denardo et al., Bowyer et al., and Rosenberg have previously looked into pediatric rheumatology diagnoses at the individual clinic and health system level, but there has been little published in the last 20 years to compare to our current study (3-5). It is hard to equate clinic volumes given multiple obscured factors like the number of providers, catchment area, etc., but compared to our median *monthly* new patient rate of 52-137 patients, these previously reported population numbers equate to an average of 71-172 new patients per clinic per *year*, demonstrating a substantial difference in patient load. The proportions of rheumatic disease diagnoses within the Denardo et al. and Bowyer et al. cohorts were reported to be 38% and 40.5%, respectively (3-4). In the Rosenberg cohort, out of 3268 patient referrals, a diagnosis was reached in only 2098 patients (64.2%), and of those diagnosed, 50.9% had rheumatic disease. Therefore, if we assume that all undiagnosed patients did not have a rheumatic disease (likely not correct), the rheumatic disease diagnosis rate of all *referred* patients would be 32.6%, with the true proportion likely higher, as some amount of the undiagnosed patients likely did have a yet-to-be-diagnosed rheumatic condition (5). Again, the comparison to our clinic’s 23.1% rheumatic disease diagnosis rate is difficult given our policy of offering appointments to all referred pediatric patients, but all previously reported cohorts had notably higher rates of rheumatic diagnoses. Juvenile rheumatoid arthritis/JIA was the most common rheumatic diagnosis in all three studies at 53%, 39.4%, and 31.6%, comparable to our JIA prevalence of 45.6% (3-5). Of the remaining non-rheumatic diagnoses, musculoskeletal conditions (56%, 36.1%) were most common, but at a smaller proportion than our 61.7% (3, 5). Therefore, despite the previously reported populations having lower total patient volume and less rheumatic disease overall, the proportions of specific rheumatic conditions within the total rheumatic diagnosis cohort seemed to be similar to our current population, with our clinic having a higher rate of non-rheumatic disease.

The pediatric rheumatology workforce supply in the United States is projected to significantly lag demand over the next few decades. As of 2018, 42 out of 50 states were noted to have less than one pediatric rheumatologist per 100,000 children and 30% of practicing pediatric rheumatologists self-reported as likely to retire in the following 10 years (2). And although there may be almost 400 pediatric rheumatologists practicing in the US and it’s likely that adult rheumatologists may see pediatric patients in various settings, the total clinical full-time equivalents (FTEs) devoted specifically to pediatric rheumatic care was reported to be 287 FTEs in 2015, even when including nurse practitioners (NPs) and physician assistants (PAs) (2). Demand for pediatric rheumatology care was estimated at 382 FTEs in 2015, already a shortfall of 95 FTEs with the 2015 workforce, and this gap is only expected to worsen by 2030 with the projected supply of 231 FTEs insufficient for the projected demand of 461 FTEs (2). Strategies have been recommended to increase the supply of pediatric rheumatology providers, including increasing exposure to the field during medical school and residency, decreasing fellowship training from 3- to 2-year commitments, increasing NP and PA utilization, and financial incentive programs (2, 6).

The demand side of the supply-demand shortfall may be a more complicated issue to address. Despite the 4-6 attending physicians, 3-4 nurse practitioners, and 1-3 pediatric rheumatology fellows that saw patients throughout our study period, it was and continues to be a struggle to see our large patient load without long appointment wait times. Moreover, even though there are limited studies focused on wait times for rheumatology evaluation, this is not a problem unique to our division. One study of adult patients referred to Ontario rheumatologists from 2000-2013 noted a median wait time from referral to rheumatologist consultation of 74 days, decreasing to 66 days for patients with systemic inflammatory rheumatic disease (7). In pediatric rheumatology, organizations in the United Kingdom and in Canada have set benchmark times for rheumatology evaluation at 4 weeks from referral for non-systemic JIA, but there is limited data on whether United States pediatric rheumatology centers can or do meet these guidelines (7, 8). During the study period, the median time between referral and appointment (wait time) for all patients was found to be 42.0 days, outside the recommended 4 weeks for rheumatology appointment wait times. However, for those patients eventually diagnosed with a rheumatic condition, the median wait time was found to be much lower at 13.8 days, well within the recommended timeframe. Wait times for individual rheumatic diagnoses were found to vary, but patients with Raynaud phenomenon were the only ones with wait times outside of 28 days. In those patients diagnosed with a non-rheumatic condition, median wait time was 49.0 days, with infection-related diagnoses (reactive arthritis, serum sickness, transient synovitis) the only category inside of 28 days. These findings seem to suggest that our providers are proficient at triaging referrals based on likelihood of rheumatic disease, recommending earlier appointments for those deemed high-risk and those at low risk receiving later appointments.

It might be prudent in our case, and in pediatric rheumatology as a whole, to focus on strategies to decrease demand for non-essential referrals, targeting those 76.9% of new patient referrals that do not have a rheumatic disease. One potential way to reduce referrals for non-rheumatic disease is to target primary care provider education. Previous studies have reported on the inappropriate ordering of laboratory testing by primary care providers, including antinuclear antibody (ANA) levels and rheumatoid factor, and the improper interpretation of musculoskeletal pain as a symptom of rheumatic disease in the pediatric population (9-10). The Choosing Wisely campaign has also previously highlighted unnecessary autoantibody panels and repeat ANA testing in its “Top 5” practices that add to the cost of care without improving quality (11). In our cohort, benign musculoskeletal pain made up 61.8% of our non-rheumatic disease diagnoses and 47.5% of all new patients seen during the study period. In the 1223 patients (46.4% of the cohort) who had musculoskeletal pain listed in the reasoning for referral to pediatric rheumatology, only 11.6% were diagnosed with a rheumatic condition. Similarly, of the 546 patients with “positive ANA” in their referral reason, either as the sole reason or in conjunction with other symptomology, 7.1% were diagnosed with rheumatic disease. By improving the ability of primary care providers to conduct musculoskeletal examinations and correctly order and interpret rheumatology laboratory testing, we may be able to limit referrals for non-rheumatic ailments.

An additional focus on the correct identification of benign musculoskeletal pain as a somatic symptom of depression and anxiety may also be helpful in reducing non-rheumatic referrals. In the last decade, numerous studies have shown a decline in the overall mental health of pediatric and adolescent patients, with significant increases in rates of depression, anxiety, and mental-health-related emergency department visits (12-13). There is a high prevalence of somatic symptoms in patients with depression and anxiety, and these patients may report only somatic symptoms at their initial primary care provider evaluation (14). Such a presentation may lead to a pediatric rheumatology referral for evaluation of potential inflammatory causes of pain. In our population, somatic disorders like AMPS and chronic fatigue syndrome were diagnosed in 227 patients from 2019-2021, making up 8.6% of all new patients seen during that period. We may be able to reduce the amount of unnecessary pediatric rheumatology referrals by targeting these few simple topics for primary care education, especially in under-resourced communities.

This study is limited by its single-center population, which makes generalizability difficult to assess, especially given our practice of offering all patients appointments regardless of the likelihood of true disease during the referral process. The COVID-19 pandemic appearing during the study period may have also altered rheumatology referral quantity and quality. In-person clinic appointments were drastically limited between February 2020 and May 2020 leading to a signficiant drop in new patient appointments. However, the limited dip in referral numbers with a rapid return to baseline levels makes this source of error unlikely. March 2021 was an outlier in terms of referral quantity that does not have such an easy explanation. Patients with non-rheumatic diagnoses doubled from just the month before while rheumatic diagnoses stayed constant. The one potential explanation that has been discussed is that Alabama saw its largest peak in COVID-19 cases in December 2020 - January 2021, so it is possible that the increase in non-rheumatic diagnoses was related to non-specific post-viral symptoms. Finally, “no-shows” of scheduled referrals and those patients diagnosed initially while inpatient were not counted in our analysis, and it is unclear how this affected the overall rates of diagnosed rheumatic disease.

**Conclusion**

As the field of pediatric rheumatology expands in its diagnostic and treatment capabilities, a serious workforce supply-demand gap has the potential to limit our ability to care for patients with rheumatic disease. As shown by our analysis and previous studies, a sizable proportion of patients referred to and evaluated in pediatric rheumatology clinics are not diagnosed with a rheumatic condition. Timely pediatric rheumatology evaluation may be achieved through the limitation of non-rheumatic disease referrals, with improved education and increased management of these conditions in the primary care space. With the supply of pediatric rheumatology providers projected to decline, intervention in referrals made to pediatric rheumatology may allow for better accessibility and quality for care for patients requiring ongoing management of a diagnosed rheumatic disease.

**Abbreviations**

COVID-19 - coronavirus disease 2019; JIA - juvenile idiopathic arthritis; FTE - full-time equivalent; IQR - interquartile range; NP - nurse practitioner; PA - physician assistant; ANA - antinuclear antibody

**Declarations**

Ethics/Consent: Data abstraction and analysis was undertaken as a Quality Improvement initiative within the University of Alabama at Birmingham Pediatric Rheumatology Division, with the goal to improve the appointment referral process and decrease appointment wait times. As such, this project did not meet the formal definition of research per the US Department of Health and Human Services and was not formally supervised by the Institutional Review Board per policy.

Consent for publication: Manuscript does not contain any specific individual’s data. Not applicable.

Availability of data and materials: The data that support the findings of this study are available from the corresponding author upon reasonable.

Competing Interests: None of the above authors report any relationships, conflicts of interest, or commercial interests specifically related to the submitted manuscript.

- RQC declares consulting fees for Sobi regarding the use of anakinra in clinical trials to treat macrophage activation syndrome; textbook royalties from Springer; speaker fees from Lilly, Sobi, MedStudy, and Arthros Rheum; and consulting or advisory fees from Sironax and CareerPhysician. RQC also serves on adjudication committees and has received personal fees to establish episodes of macrophage activation syndrome among patients enrolled in clinical trials (funded by Pfizer and AbbVie) for therapeutics used to treat juvenile idiopathic arthritis. RQC holds a patent on detecting rare long non-coding RNA mutations in people with COVID-19 unrelated to the content of this report.
- EAS and MLM are both supported by the Rheumatology Research Foundation Investigator Award.
- DDR, JMB, ECR, VLG, LM, AR, CS, BW, PW, and MLS declare no conflicts of interest.

Funding/Support: No funding was secured for this study.

Authors’ contributions: DDR contributed to conceptualization/design, methodology, investigation, data curation, formal analysis, and writing/editing the manuscript. JMB, ECR, VLG, LM, AR, CS, BW, PW, and MLS contributed to data curation and reviewing/editing of the manuscript. EAS, MLM, and RQC contributed significantly to conceptualization/design, supervision/oversight, and reviewing/editing the manuscript. All authors have read and approved of the final manuscript in its entirety.

Acknowledgement: Not Applicable.

**References**

1. Brewer EJ. A peripatetic pediatrician's journey into pediatric rheumatology. Pediatr Rheumatol Online J. 2007;5:11. Published 2007 May 23. doi:10.1186/1546-0096-5-11
2. Correll CK, Ditmyer MM, Mehta J, et al. 2015 American College of Rheumatology Workforce Study and Demand Projections of Pediatric Rheumatology Workforce, 2015-2030. Arthritis Care Res (Hoboken). 2022;74(3):340-348. doi:10.1002/acr.24497
3. Denardo BA, Tucker LB, Miller LC, Szer IS, Schaller JG. Demography of a regional pediatric rheumatology patient population. Affiliated Children's Arthritis Centers of New England. J Rheumatol. 1994;21(8):1553-1561.
4. Bowyer S, Roettcher P. Pediatric rheumatology clinic populations in the United States: results of a 3 year survey. Pediatric Rheumatology Database Research Group. J Rheumatol. 1996;23(11):1968-1974.
5. Rosenberg AM. Longitudinal analysis of a pediatric rheumatology clinic population. J Rheumatol. 2005;32(10):1992-2001.
6. Argraves M, Mehta JJ. Pediatric rheumatology: Not just a little adult workforce issue. Semin Arthritis Rheum. 2021;51(5):e13. doi:10.1016/j.semarthrit.2020.12.005
7. Widdifield J, Bernatsky S, Thorne JC, et al. Wait times to rheumatology care for patients with rheumatic diseases: a data linkage study of primary care electronic medical records and administrative data. CMAJ Open. 2016;4(2):E205-E212. Published 2016 May 11. doi:10.9778/cmajo.20150116
8. Davies K, Cleary G, Foster H, Hutchinson E, Baildam E; British Society of Paediatric and Adolescent Rheumatology. BSPAR Standards of Care for children and young people with juvenile idiopathic arthritis. Rheumatology (Oxford). 2010;49(7):1406-1408. doi:10.1093/rheumatology/kep460
9. Correll CK, Spector LG, Zhang L, Binstadt BA, Vehe RK. Use of Rheumatology Laboratory Studies Among Primary Pediatricians. Clin Pediatr (Phila). 2016;55(14):1279-1288. doi:10.1177/0009922815624902
10. McGhee JL, Burks FN, Sheckels JL, Jarvis JN. Identifying children with chronic arthritis based on chief complaints: absence of predictive value for musculoskeletal pain as an indicator of rheumatic disease in children. Pediatrics. 2002;110(2 Pt 1):354-359. doi:10.1542/peds.110.2.354
11. Rouster-Stevens KA, Ardoin SP, Cooper AM, et al. Choosing Wisely: the American College of Rheumatology's Top 5 for pediatric rheumatology. Arthritis Care Res (Hoboken). 2014;66(5):649-657. doi:10.1002/acr.22238
12. Lebrun-Harris LA, Ghandour RM, Kogan MD, Warren MD. Five-Year Trends in US Children's Health and Well-being, 2016-2020 [published correction appears in JAMA Pediatr. 2022 Apr 4;:null] [published correction appears in JAMA Pediatr. 2023 Mar 1;177(3):323]. JAMA Pediatr. 2022;176(7):e220056. doi:10.1001/jamapediatrics.2022.0056
13. Bommersbach TJ, McKean AJ, Olfson M, Rhee TG. National Trends in Mental Health-Related Emergency Department Visits Among Youth, 2011-2020. JAMA. 2023;329(17):1469-1477. doi:10.1001/jama.2023.4809
14. Simon GE, VonKorff M, Piccinelli M, Fullerton C, Ormel J. An international study of the relation between somatic symptoms and depression. N Engl J Med. 1999;341(18):1329-1335. doi:10.1056/NEJM199910283411801

**Data Supplements:** None

**Appendix:** None
